# RMaNI: Regulatory Module Network Inference framework

**DOI:** 10.1186/1471-2105-14-S16-S14

**Published:** 2013-10-22

**Authors:** Piyush B Madhamshettiwar, Stefan R Maetschke, Melissa J Davis, Mark A Ragan

**Affiliations:** 1 Institute for Molecular Bioscience, The University of Queensland, 306 Carmody Road, St Lucia, Brisbane, Queensland 4072, Australia; 2 Australian Research Council Centre of Excellence in Bioinformatics, The University of Queensland, 306 Carmody Road, St Lucia, Brisbane, Queensland 4072, Australia

**Keywords:** Cancer, Systems biology, Transcriptional Module Networks, Microarray, Gene Regulatory Network, Modules

## Abstract

**Background:**

Cell survival and development are orchestrated by complex interlocking programs of gene activation and repression. Understanding how this *gene regulatory network *(GRN) functions in normal states, and is altered in cancers subtypes, offers fundamental insight into oncogenesis and disease progression, and holds great promise for guiding clinical decisions. Inferring a GRN from empirical microarray gene expression data is a challenging task in cancer systems biology. In recent years, module-based approaches for GRN inference have been proposed to address this challenge. Despite the demonstrated success of module-based approaches in uncovering biologically meaningful regulatory interactions, their application remains limited a single condition, without supporting the comparison of multiple disease subtypes/conditions. Also, their use remains unnecessarily restricted to computational biologists, as accurate inference of modules and their regulators requires integration of diverse tools and heterogeneous data sources, which in turn requires scripting skills, data infrastructure and powerful computational facilities. New analytical frameworks are required to make module-based GRN inference approach more generally useful to the research community.

**Results:**

We present the RMaNI (Regulatory Module Network Inference) framework, which supports cancer subtype-specific or condition specific GRN inference and differential network analysis. It combines both transcriptomic as well as genomic data sources, and integrates heterogeneous knowledge resources and a set of complementary bioinformatic methods for automated inference of modules, their condition specific regulators and facilitates downstream network analyses and data visualization. To demonstrate its utility, we applied RMaNI to a hepatocellular microarray data containing normal and three disease conditions. We demonstrate that how RMaNI can be employed to understand the genetic architecture underlying three disease conditions. RMaNI is freely available at http://inspect.braembl.org.au/bi/inspect/rmani

**Conclusion:**

RMaNI makes available a workflow with comprehensive set of tools that would otherwise be challenging for non-expert users to install and apply. The framework presented in this paper is flexible and can be easily extended to analyse any dataset with multiple disease conditions.

## Background

Complex cellular behaviour in cancer is orchestrated by the action of transcriptional regulatory networks [1,2]. Computational inference of transcriptional regulatory networks, referred to as Gene Regulatory Networks (GRN), from microarray gene expression data is one of the fundamental goals of systems biology and its translation to genomic medicine [3]. GRN inference and analysis, especially when integrated with experimental validation, has proven to be a powerful tool in understanding how regulatory networks are disrupted and rewired in normal and cancer conditions, and in identifying novel regulatory interactions as well as broader systemic disruptions in key oncogenic processes [4-6]. Many methods have been developed to infer GRNs from microarray gene expression data. These approaches include unsupervised, semi-supervised and supervised methods based on computational mathematics, multivariate statistics and information science [7-11].

Although diverse computational and statistical approaches have been applied to this problem, the accuracy of edge-wise network inference methods remains poor [11-14]. Novel approaches are needed to address the genome-wide network inference problem. A promising direction is the inference of transcriptional modules instead of individual edges. Module inference is simpler than edge-wise network inference [15,16], and higher accuracies can be achieved [7,17].

### Transcriptional module networks

Several studies have revealed that regulatory networks are modular in nature and organised hierarchically [18]. According to Oltvai and Barabasi's "complexity of life" pyramid, functional modules are less complex compared to individual transcriptional programs, which in turn are the building blocks for these modules [15]. Therefore, inferring modules instead of the individual interactions of complete networks drastically reduces the complexity of the inference problem, and shows great promise for network analysis in complex disease conditions including cancer [17,19-21]. A transcriptional-module network is composed of clusters of co-expressed genes collaboratively or alternatively regulated by one or several transcription factors (TFs) *via *convergent or divergent regulatory programs. A *convergent regulatory program *represents a particular set of target genes (TGs) regulated by different sets of TFs, whereas a *divergent regulatory program *represents a given set of TFs regulating distinct sets of TGs [7,22].

Several methods have been developed to infer modules from microarray data, including a range of clustering methods such as *k*-means, hierarchical clustering and self-organizing maps. However, all these approaches suffer from certain limitations; for instance, the number of clusters is not determined automatically but requires the number of clusters to be pre-specified [23-26]. WGCNA [27], based on the *weighted gene co-expression network analysis *approach [28], is the most widely used method and has been applied to a number of diseases [29-32]. It also uses a clustering approach to infer modules, but it optimizes the threshold to achieve a scale-free topology. Assuming scale-freeness, several model-based clustering approaches have been developed [33-35]. Model-based approaches allow a statistical analysis of the inferred modules and automatically estimate the number of modules [34]. For example, Genomica [20] uses expectation maximisation (EM) to identify modules [16,20].

Other methods [22,36-39] use additional experimental data such as protein-protein interactions, TF binding affinity data, *in vitro *DNA binding specificities, DNA motifs and ChIP-chip data. Such integrative approaches are attractive and promising approaches to infer modules, as they take into account different sources of biological information [40]. However, they do not natively integrate methods for module inference, identification of regulators, or comprehensive downstream analysis and visualization. Also, they support the analysis only of individual datasets arising from only one condition without differential analysis of other conditions or subtypes.

Integrating diverse data sources as well as multiple methods brings many challenges. These challenges can be diverse, range from methodological to practical in nature, and can arise due to the computational or statistical complexities of methods and the dimensionality of omic data [41,42]. For instance, combining heterogeneous data requires extensive file formatting at different stages of analysis, while integrating different methods involves the selection or optimization of diverse parameters and other user-control features. As a consequence of these challenges, it is difficult for biologists or clinicians (without strong informatic skills) to chain multiple methods together into comprehensive, flexible workflows to address substantial questions. For example, to identify the modules involved in any disease condition one must retrieve data from different repositories (*e.g*. motif data from Transfac [43] or Genomatix [44]), map the identifiers *e.g*. using Biomart [45], perform differential gene expression analysis *e.g *using LIMMA [46], infer the modules and identify regulators *e.g*. using Genomica [20], integrate the inferred modules and regulators for visualization *e.g*. using Cytoscape [47], and finally perform functional analysis of module genes *e.g*. using DAVID [48,49]. This work focuses on making available a workflow and computational resources for the inference of modules and their regulators, downstream analyses and visualization.

### RMaNI - Regulatory Module Network Inference framework

Here, we present a novel integrative and automated analytical framework "RMaNI - Regulatory Module Network Inference" for disease condition or subtype-specific module network inference, analysis and data visualization. It uses the Learning Module Networks (LeMoNe) algorithm [50] and Regulatory Impact Factors (RIF) [51] to identify relevant regulatory TFs. The LeMoNe algorithm uses a Bayesian probabilistic model-based approach for clustering genes, and in selecting thresholds does not assume that networks necessarily have a scale-free topology [50].

RMaNI combines both transcriptomic as well as genomic data sources, and integrates heterogeneous knowledge resources and a set of complementary bioinformatic methods for microarray data processing, differential expression (DE) analysis, module detection and regulator identification, gene and module significance measure calculations, functional enrichment analysis of module genes, and visualization of data and networks.

### Case study - application to hepatocellular carcinoma

To demonstrate its utility, we applied RMaNI to a hepatocellular microarray dataset containing normal tissue and three disease conditions: pre-malignant (cirrhosis), cirrhosis with hepatocellular carcinoma (cirrhosisHCC), and hepatocellular tumor (HCC). We illustrate that the identification and analysis of transcriptional module network can give insight into the common and unique genetic architecture underlying hepatocellular carcinoma conditions.

## Implementation

The RMaNI web interface has been created using Rwui [52], a Java-based application that uses the Apache Struts framework. The complete application is running on a Tomcat server on a high-performance computing cluster. The workflow integrates publicly available R[53], Bioconductor [54] and custom packages and functions for data import, processing, analysis, integration and visualization. All packages are currently running under R version 2.15.2, and can be easily updated as newer versions of R are released. RMaNI is freely available as a user-friendly web-application at http://inspect.braembl.org.au/bi/inspect/rmani, with a comprehensive manual available (Additional File 1).

In the next section, we describe the RMaNI workflow and provide a brief overview of the methods used in each step. Then, we present a case study showing how RMaNI can be employed to understand the genetic architecture underlying three hepatocellular carcinoma conditions.

## RMaNI: structure and functionalities

**Figure 1:**
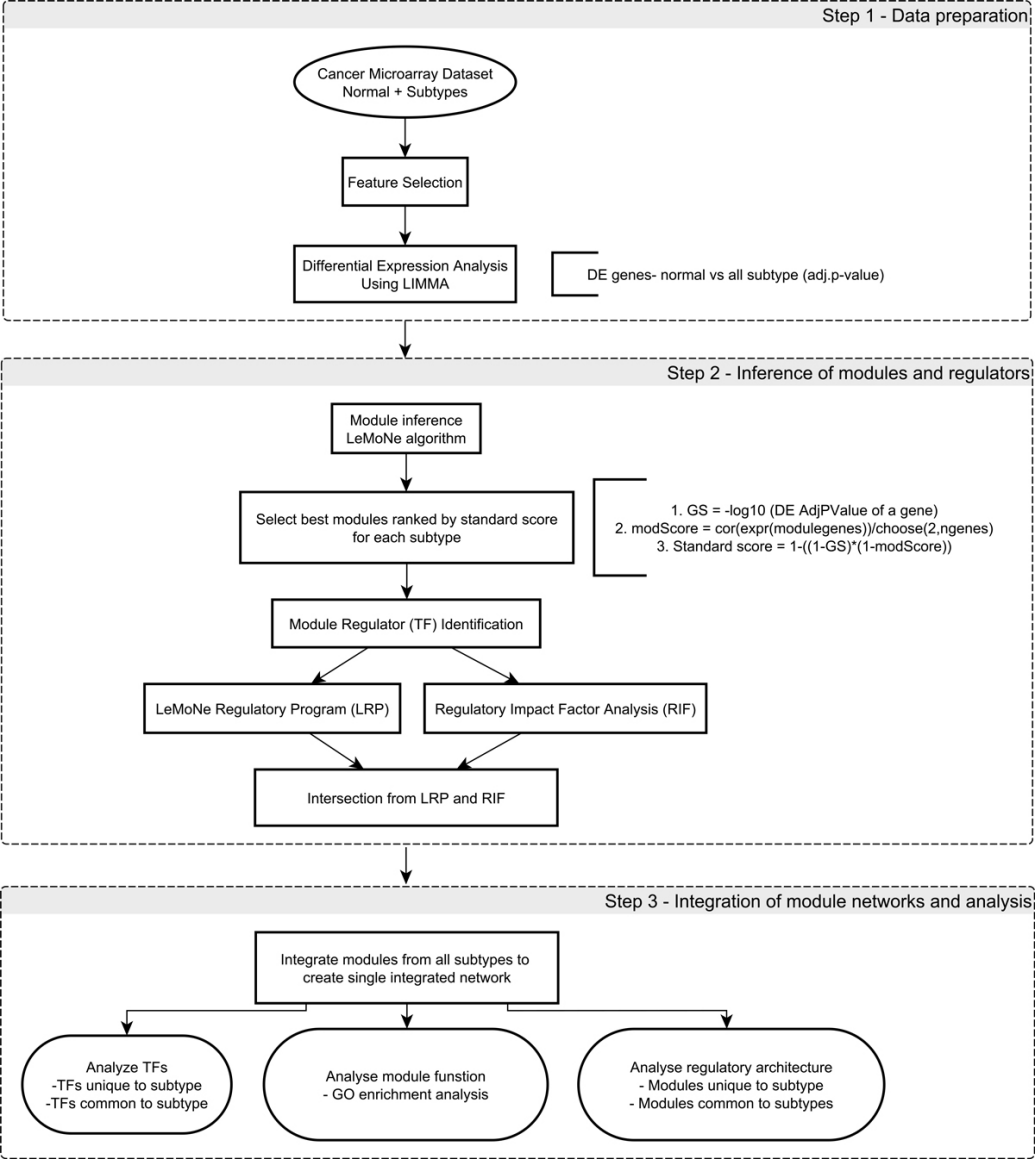
**RMaNI workflow**. Stages involved in RMaNI workflow. Workflow is divided into three main stages - Data preparation, Inference of modules and regulators, and Integration of module networks and analysis.

Figure 1 illustrates the workflow in RMaNI. The workflow is divided into three main stages: 1) data preparation, 2) inference of modules and regulators, and 3) integration of module networks and analysis. In this section, we describe these stages and the individual steps involved therein.

### Stage 1 - Data Preparation

At this stage, the pre-processed (background corrected and normalized) microarray gene expression data and sample annotations are imported from files uploaded by the user.

### Step 1.1 - Dataset

RMaNI can be applied to gene expression datasets arising from multiple conditions. Currently, we support datasets arising from 13 different types of Affymetrix chips: hgu133a, hgu133a2, hgu133b, hgu133plus2, hgu219, hgu95a, hgu95av2, hgu95b, hgu95c, hgu95d, hgu95e, hthgu133a and hthgu133b.

### Step 1.2 - Feature selection for input to module inference workflow

Once a user has the microarray dataset, the question arises: which and how many features should one input to the network inference step? Because there is no standard feature-selection method or recommendation on the minimum number of features, the workflow compares different feature-selection methods for different gene sets, and identifies the optimal combination of these two parameters.

The user compares three feature-selection methods: differentially expressed genes between normal and all subtypes (DE_all), differentially expressed genes between normal and each subtype (DE_pair), and the most-variable genes across the dataset based on the coefficient of variation (Var). For differential expression analysis RMaNI uses the LIMMA package [46], and to select variable genes it uses a custom R function. To find the optimal number of genes, for each of the three feature selection methods, it selects eight subsets with 10 to 4000 genes (10, 50, 100, 200, 500, 1000, 2000 and 4000 genes) optimal for network inference step. To identify the optimal feature selection method and number of genes, it examines how well they group the samples into the known classes.

The user compares the different gene sets on seven different clustering methods (clues, kmeans, PAM, AGNES, Fanny, SOTA and MCLUST) [55-57]. The workflow uses the Rand Index (RI) [58] as a measure for evaluating the clustering performance. RI measures the similarity between two data clusterings (known against predicted). An RI equal to 1 indicates perfect clustering, while an RI of 0 indicates that the clustering is no better than chance. These methods are implemented in the R packages clValid [59], clues [55], cluster [57] and mclust [56]. A brief description of each clustering method is given below.

**clues **(clustering based on local shrinking) is a nonparametric clustering method using local shrinking [55]. It estimates the number of clusters and simultaneously finds a partition of a data set *via *three steps: shrinking, partition, and determination of the optimal number of partitions.

**kmeans **is a parametric, centroid-based clustering method. Given the number of clusters, it starts with an initial estimate for the cluster centroids, and each sample is assigned to the cluster with the nearest mean [60]. The cluster centroids are then updated, and the entire process is iterated until the cluster centroids become stable.

**PAM **(Partitioning Around Medoids) is a parametric method similar to *k*-means, but PAM is a medoid-based method. A *medoid *is a representative object of a cluster, such that its average dissimilarity to all objects in that cluster is minimal [61]. Given the number of clusters, PAM starts with an initial estimate for the cluster medoids, and calculates the dissimilarity matrix using the Euclidean or Manhattan distance [61]. Based on this matrix, each sample is assigned to the cluster with the nearest medoid.

**AGNES **(AGglomerative NESting) is a hierarchical clustering method which groups a dataset into a tree of clusters [61]. It is a bottom-up clustering method that starts with small clusters of single samples and then, at each step using a specified distance metric, merges the clusters into larger cluster. This is repeated iteratively until a single cluster is obtained, containing all samples.

**Fanny **is a fuzzy or soft clustering method [61]. With this method each sample has partial membership with each cluster rather than belonging exclusively to just a single cluster. Each sample describes the probability scores for its cluster membership. After optimizing the number of clusters, the method starts with assigning random cluster probabilities to each sample, and repeats this process until convergence.

**SOTA **(Self-Organising Tree Algorithm) is a divisive clustering method [62]. It generates an unsupervised neural network with a binary tree topology. Contrary to AGNES, SOTA is a top-down clustering method. It starts the clustering process with a binary tree consisting of a root node with two leaves, each representing one cluster. The self-organizing process then grows the tree by converting the leaf with the highest score into a node and attaching two new leaves to it. The score for each cluster is defined as the mean value of the distances between the cluster and the samples associated with it [63].

**MCLUST **(Model based clustering) is a nonparametric, model-based clustering method that uses finite normal mixture modelling and the expectation maximisation (EM) algorithm. Unlike other methods, it does not require the number of clusters as input, but instead infers the number of clusters from the data.

In summary, stage 1 provides an estimate on the feature-selection method and optimal number of genes which best explains the given data. The user can choose this feature selection method and this many genes for input to find clusters of co-expressed genes in the next step. For ease and flexibility of processing the user's own data, the feature selection step is not supported through the RMaNI web-interface.

### Stage 2 - Clustering of genes to modules and identification of regulators

This is the main stage of the RMaNI workflow. It takes the gene set optimized in the feature selection step, and uses the corresponding gene expression data for module network inference. Below we provide the details of the individual steps.

### Step 2.1 - Inference of transcriptional module networks

Given a gene expression dataset and a set of candidate regulators (TFs, microRNA or clinical variable of interest like stage or grade); inference of modules is composed of two steps: first clustering of co-expressed genes to identify modules, and second the inference of links between regulators and modules.

### Step 2.1.1 - Clustering of genes

RMaNI uses the LeMoNe (Learning Module Networks) algorithm for inferring modules from microarray data, LeMoNe performs a two-way Bayesian clustering of genes and uses a Gibbs sampling procedure to iteratively update the cluster assignments of genes [34,50]. Each inferred module contains the genes for which the expression profiles best fit the same multivariate normal distribution [7]. LeMoNe has been successfully applied to different conditions including cancer [17,64-67]. LeMoNe outputs the ensemble of clustering solutions represented as a gene-to-cluster probability matrix reflecting the probability of the assignment of a gene to each module, referred to as fuzzy clustering (one gene can belong to multiple modules, each with certain probability). Using a graph spectral method and a probability cut-off, it then outputs tight clusters (in which one gene belongs to only one cluster) from fuzzy clusters [50].

### Step 2.2 - Inferring the regulators

To identify and prioritize potential TFs regulating modules, candidate TFs are gathered by integrating lists of TFs from Vaquerizas [68], Ravasi [69], TCOF-DB [70] and Transfac [43]. To infer the potential regulator for each module, two methods are employed: LeMoNe's regulatory program (LRP) and the Regulatory Impact Factor analysis (RIF) algorithm. Below, we briefly describe these methods.

### Step 2.2.1 - LeMoNe regulatory program

In LeMoNe's regulatory program, two types of regulators can be assigned, regulators with continuous or with discrete values. Continuous values include expression values measured, for example, for TFs, signal transducers, kinases and/or microRNAs. Discrete values can be clinical variables like tumor stage or grade. In this workflow the focus is on TFs. Transcriptional regulatory programs are inferred using a hierarchical decision-tree model. The regulator assigned to each module consists of the set of TFs for which the expression profiles best explain all or part of the conditions. TFs receive a regulatory score reflecting the statistical confidence with which a TF regulates genes in the cluster. The collection of the regulatory scores for each TF is then converted into a global score. Finally, the TFs are sorted by their scores to construct a ranked list of potential regulators.

### Step 2.2.2 - Regulatory Impact Factor (RIF) analysis

RIF analysis was initially developed to identify TFs that contribute to the differential expression in a particular condition, although the TF itself is not differentially expressed [51]. RIF is based on the differential correlation between a TF and the genes differentially expressed (DE) under two conditions. To compute a regulatory confidence score, it integrates three sources of information into a single measure: (a) the change in correlation between the TF and the DE genes, referred to as differential wiring; (b) the amount of differential expression of DE genes; and (c) the abundance of DE genes under the two conditions. It assigns a score (RIF1) to those TFs that are consistently most differentially co-expressed with the highly abundant and highly expressed DE genes, and another score (RIF2) to those TFs with the most altered ability to predict the abundance of DE genes [51].

### Step 2.3 - Gene significance measures for module ranking

RMaNI uses two measures to rank the modules for each subtype, average gene significance (GS) and modScore. The average gene significance focuses on the differential expression of genes in two conditions in each module and the modScore represents the overall correlation between genes in each module. RMaNI combines these two scores into a single score referred as a standard score:

averageGS = average(-log10(DE pvalue of a gene))

modScore = sum(abs(correlation of genes))/choose(ngenes,2))

standardScore = 1 - ((1 - averageGS])*(1 - modScore))

### Stage 3 - Integration of transcriptional module networks and topological analysis

At this stage, the workflow combines all subtype-specific modules and regulators to build a transcriptional module network. In the topological analysis of such a module network, RMaNI calculates the overlap of TFs, TGs and interactions across subtypes, and generates node and edge attributes to aid in visualization.

### Step 3.1 - Functional enrichment analysis of the inferred modules

The genes in each of the modules are subjected to a functional GO enrichment analysis using BiNGO [71]. Significantly enriched GO terms are detected by a hypergeometric test with adjusted Benjamini-Hochberg False Discovery Rate (FDR) [72] correction at significance level 0.05 against the all other genes in the network as a background.

### Step 3.2 - Cluster similarity measures

To visualize the similarities between different modules, RMaNI uses the Jaccard similarity index as an external measure and Biological Process (BP) and Molecular Function (MF) as biological measures. The Jaccard index is calculated as the number of unique genes common to two clusters divided by the total number of unique genes in two sets. The BP and MF similarity measures are calculated by the GOSemSim package in Bioconductor [73].

### Step 3.3 - Visualization

Throughout the analysis a number of figures are generated for data visualization, including the representation of inferred modules, significance measures calculated for each module, and overlaps of TFs, TGs and interactions across all subtypes. To visualize the network, the workflow exports interactions to a Cytoscape [47] -compatible file. Node attributes such as subtype and module memberships, number of modules regulated by a TF, GO annotations, and edge attributes such as subtype membership of an interaction, and regulatory score for an edge, are also provided for further exploration.

## Application of RMaNI to hepatocellular carcinoma

To demonstrate the utility of RMaNI, we applied this workflow to hepatocellular carcinoma dataset (GSE14323) [74], containing normal tissues and three disease conditions: pre-malignant (cirrhosis), cirrhosis with hepatocellular carcinoma (cirrhosisHCC), and hepatocellular tumor (HCC). We investigated the ability of RMaNI to infer condition-specific transcriptional module networks, find common and unique TFs and regulatory interactions to examine the genetic architecture and ultimately to understand the differences and similarities between conditions. Below, we present the results for the individual steps to demonstrate the workflow.

### Dataset

We used a Robust Multiarray Averaging (RMA) normalised and standardised hapatocellular carcinoma microarray gene expression dataset, based on 115 samples (Table 1).

**Table 1:** Description of the hepatocellular carcinoma microarray dataset.

Dataset	No. of samples in each condition				Platform
		
	Normal	Cirrhosis	CirrhosisHCC	HCC	
GSE14323 115 samples	19	41	17	38	HG-U133A (12079 probes)

In the next step, we input dataset to the LeMoNe algorithm to infer modules.

### Inference of module networks in hepatocellular carcinoma conditions

We selected top 4000 differentially expressed genes (based on BH-adjusted p-value) between normal and three conditions to infer the modules as described in the workflow. For this step RMaNI uses the LeMoNe algorithm. Michoel et al. [65] evaluated performance of the LeMoNe against state-of-the-art method *genomica*, and Smet and Marchal [7] compared LeMoNe against other network inference methods. For each pair of the normal-to-condition datasets (Table 2), 10 clustering solutions were generated. For each run LeMoNe used the default setting of 50 burn-ins and 100 Gibbs sampling steps, where the minimum number of genes in a cluster was set to 4. The default probability score cut-off of 0.2 was used uniquely assign genes to clusters.

**Table 2:** Summary of the datasets used in the study, five sets of normal and subtype pairs data were input to LeMoNe.

Datasets	No. of DE Genes	No. of Samples
Normal + cirrhosis	4000	60

Normal + cirrhosisHCC	4000	36

Normal + HCC	4000	57

Table 3 summarizes the clustering results. For each condition, it shows the different number of clusters generated, with their number of genes, maximum and minimum module sizes. We also performed a GO enrichment analysis on each module using BiNGO to measure the functional coherence of genes in the modules. Table 3 also shows the total number of modules, in each subtype, with at least one significant GO category enriched (BH-adjusted p-value 0.05). For instance, in cirrhosis, RMaNI generated a set of 74 modules corresponding with a total of 3794 genes. The largest modules had 302 genes and the smallest 4. In the next step we identified the regulators of the modules.

**Table 3:** Summary of gene clustering results.

Conditions	No. of Modules	No. of Genes	Max Module Size	Min Module Size
Normal + cirrhosis	74	3794	302	4

Normal + cirrhosisHCC	59	3813	342	4

Normal + HCC	78	3772	219	4

### Identification and ranking of regulators

To assign the potential regulators (TFs) to the inferred modules, two data-driven approaches were employed in RMaNI: LRP and RIF. This step resulted in the potential regulatory TFs ordered according to their LRP and RIF score. To find the most confident regulators for each cluster, RMaNI used the intersection of regulators identified by both methods and integrated both scores into one score (stdScore). Table 4 presents the TFs predicted that have a regulatory role in at least two conditions. For instance, it reveals that TF CBFB regulates at least one module in each of the cirrhosis, cirrhosisHCC and HCC conditions and has 557 interactions across the three conditions. Previous studies were limited to a set of prior candidate TFs only, e.g. differentially expressed TFs or TFs involved in a particular pathway but considering the fact that the detection of DE TFs from expression data is limited due to their low and sparse expression levels, RMaNI uses all the TFs of a species (human in this study) without the need of prior TF identification. However, its applicability in organisms without known TFs will largely be determined by the entirety of TF databases and annotations, which are expected to improve over time with advances in ChIP-chip and ChIP-seq studies. Other continuous regulatory factors such as microRNAs, signal transducers, kinases and discrete regulatory factors such as clinical parameter, e.g. stage, grade or treatments can also be used.

**Table 4:** TFs that are predicted to have a regulatory role in at least two conditions.

TFs	Conditions	TGs in cirrhosis	TGs in cirrhosisHCC	TGs in HCC	Total Edges
CBFB	cirrhosis, cirrhosisHCC, HCC	144	342	71	557

TCF4	cirrhosis, HCC	144	0	71	215

USF2	cirrhosis, cirrhosisHCC	144	342	0	486

Remaining TFs are unique to individual conditions.

### Identification of modules with the highest DE and correlation

To identify the modules with high DE as well as high correlation for each condition (referred as best modules), we ordered the standard score, generated from averagGS and modScore, and for each module the workflow detected the knee-point (the maximum inflection point of a graph) from standard score to select the best modules. Table 5 shows the total number of best modules selected for each condition and the number of TFs and target genes in selected modules. For instance, in cirrhosis, 7 modules corresponding to 200 genes were selected. The 200 genes include 191 TGs and 9 TFs.

**Table 5:** Summary of the modules with highest DE and correlation (best modules).

Conditions	Total Modules	No. of best Modules	No. of Genes	No. of TFs	No. of TGs
cirrhosis	74	7	200	9	191

cirrhosisHCC	59	6	183	11	172

HCC	78	6	255	30	225

Total	211	18	638	50	588

Unique	211	50	548	47	548

Best modules were selected, for each condition, from all the modules inferred.

### Network analysis

**Figure 2:**
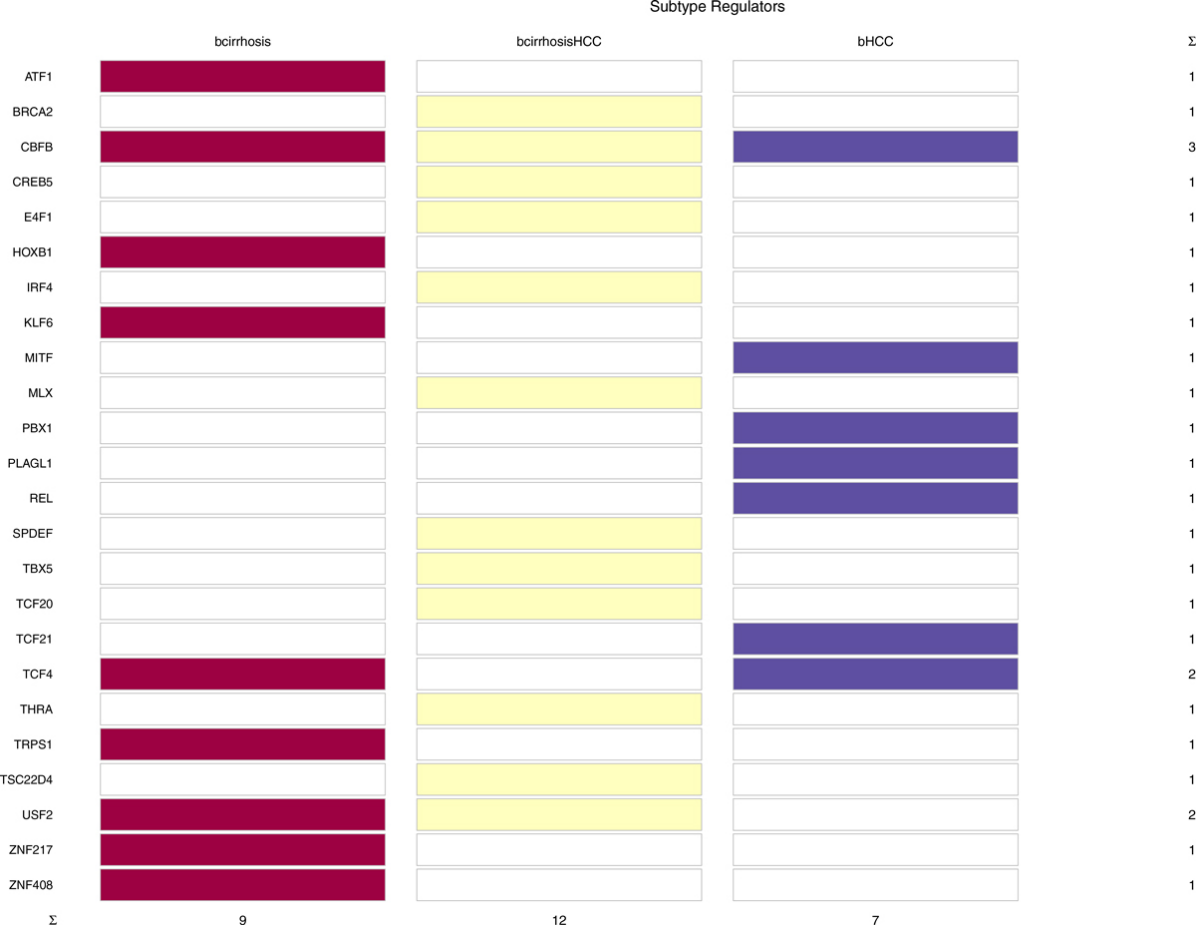
**TF overlap analysis result**. Figure illustrates TF overlap analysis results. Three TFs are predicted to have a regulatory role in at least two conditions. Remaining TFs are unique to individual conditions.

We aggregated all the module networks inferred for each condition to construct an overall network. For this purpose, RMaNI generates the network around the regulators predicted with highest confidence according to stdScore. The generated hepatocellular carcinoma network includes 24 TFs and 557 TGs connected by 5897 edges. We found 144 nodes unique to cirrhosis, 342 nodes unique to cirrhosisHCC, and 71 nodes unique to HCC. 1296, 4104 and 497 edges were unique to cirrhosis, cirrhosisHCC and HCC conditions, respectively. Previous approaches do not identify unique or shared TFs between modules, and between subtypes or conditions. By contrast, in this analysis we performed the analysis of convergent and divergent regulatory programs via TF overlap analysis. Figure 2 illustrates the TFs overlap across three conditions. We found one TF (CBFB) associated with all the three conditions, two TFs (TCF4 and USF2) associated with two conditions (Table 4) and 21 TFs were unique to one condition.

### Network visualization

**Figure 3:**
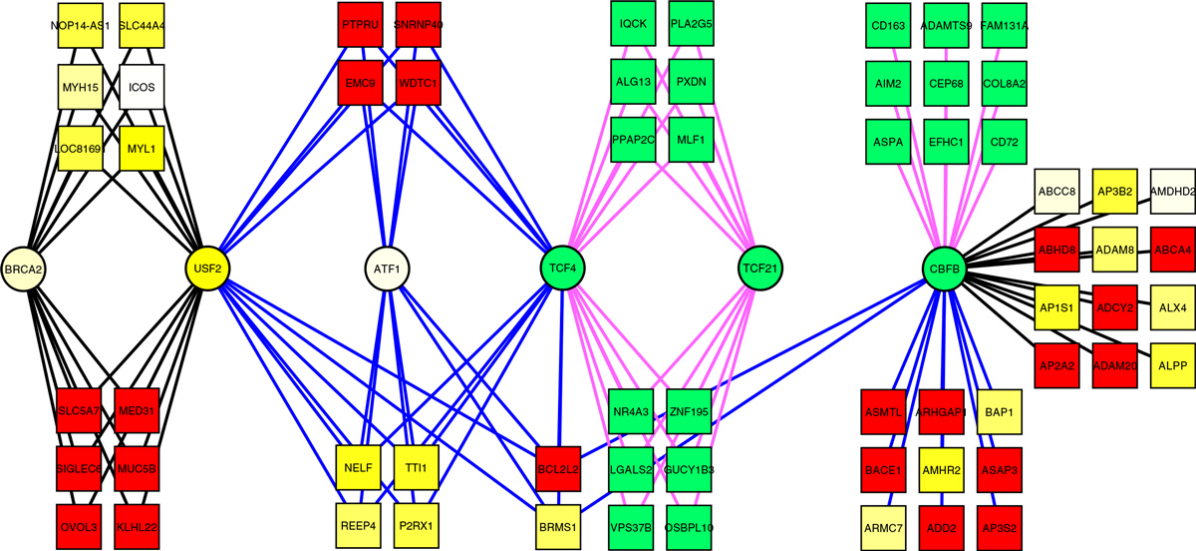
**Hepatocellular carcinoma transcriptional sub-network**. The hepatocellular carcinoma sub-network showing TGs (rectangles) and TFs (circles). Node colour gradient: red, up-regulation; green, down-regulation; yellow, no-change. Edge colors: blue, cirrhosis; black, cirrhosisHCC, cyan, HCC.

We imported the inferred module network in Cytoscape for visualization and exploration. For demonstration of topological analysis of inferred network, we extracted a sub-network of 70 nodes (TFs and TGs). Figure 3 shows hepatocellular carcinoma sub-network which includes 6 TFs and 64 TGs connected by 110 edges. Nodes and edges are rendered as per different evidences. For instance, node shape represents its type (TF or TG), and node colour gradient represents DE in cirrhosis against normal tissues. Edge colour represents condition membership. Figure shows distinct modules regulated by different TFs. It also illustrates the TF overlap analysis result (Table 4), for instance, CBFB, TCF4 and USF2 regulates the TGs in at least two conditions whereas TCF21, ATF1 and BRCA2 are predicted to have regulatory role only in one of the conditions.

## Conclusions

We have presented the RMaNI workflow, developed for the end-user perspective of a biologist or clinician. It provides an easy-to-use interface to a comprehensive, integrated suite of tools for the inference of condition or subtype-specific transcriptional module networks and their analysis. We described the RMaNI workflow and applied it to hepatocellular carcinoma data. We demonstrated that identifying the transcriptional module network, and analysing and visualizing the inferred network, can give insight into the common as well as unique regulatory architecture underlying different disease conditions. We anticipate integrating additional tools and workflows in future to meet the distinct needs of researchers confronting the complexity of cancer.

## List of abbreviations used

TF: Transcription Factor; TG: Target Gene; LeMoNe: Learning Module Networks; RIF: Regulatory Impact Factors; clues: clustering based on local shrinking; PAM: Partitioning Around Medoids; AGNES: AGglomerative NESting; SOTA: Self-Organising Tree Algorithm; MCLUST: Model based clustering; LRP: LeMoNe's regulatory program; averageGS: average Gene Significance; GO: Gene Ontology.

## Competing interests

The authors declare that they have no competing interests.

## Authors' contributions

PBM developed the RMaNI framework, wrote the code and the manuscript. SRM, MJD, MAR advised on design and features of RMaNI, provided overall scientific and technical guidance, and assisted with the manuscript. All authors read and approved the final manuscript.

## Supplementary Material

Additional File 1 RMaNI User Manual Click here for file

## Declarations

Publication of this article was funded by The University of Queensland.

This article has been published as part of *BMC Bioinformatics *Volume 14 Supplement 16, 2013: Twelfth International Conference on Bioinformatics (InCoB2013): Bioinformatics. The full contents of the supplement are available online at http://www.biomedcentral.com/bmcbioinformatics/supplements/14/S16.
