# Supplementary material for: RMaNI: Regulatory Module Network Inference framework
Source: BMC Bioinformatics. 2013 Oct 22;14(Suppl 16):S14. doi: 10.1186/1471-2105-14-S16-S14 (PMC3853211; doi:10.1186/1471-2105-14-S16-S14)
Supplement: Additional File 1 — RMaNI User Manual [file 1471-2105-14-S16-S14-S1.PDF]

RMaNI: Regulatory Module Network Inference Framework

Piyush B. Madhamshettiwar, Stefan R. Maetschke, Melissa J. Davis, and Mark A. Ragan

User Manual

RMaNI is the novel analytical workflow developed for cancer subtype or condition specific transcriptional module network inference and analysis. It uses the Learning Module Networks (LeMoNe) algorithm for model based co-clustering of the expression data and Regulatory Impact Factors (RIF) to identify potential regulators of the inferred modules. We provide a very simple web interface for complex analysis workflow. Please refer to manuscript for detailed workflow. In this user manual, we provide step-by-step instruction for analysing your data using RMaNI. The user manual is structured as follows. We first present the RMaNI homepage and access URL. Next, we provide step-by-step instructions on input data preparation, parameters required in each field and submission of analysis. Finally, we present job submission confirmation, progress and job results sections, respectively.

I. RMaNI Homepage

Figure 1 shows screenshot of the RMaNI homepage. RMaNI can be accessed via this URL - http://inspect.braembl.org.au/bi/inspect/rmani.

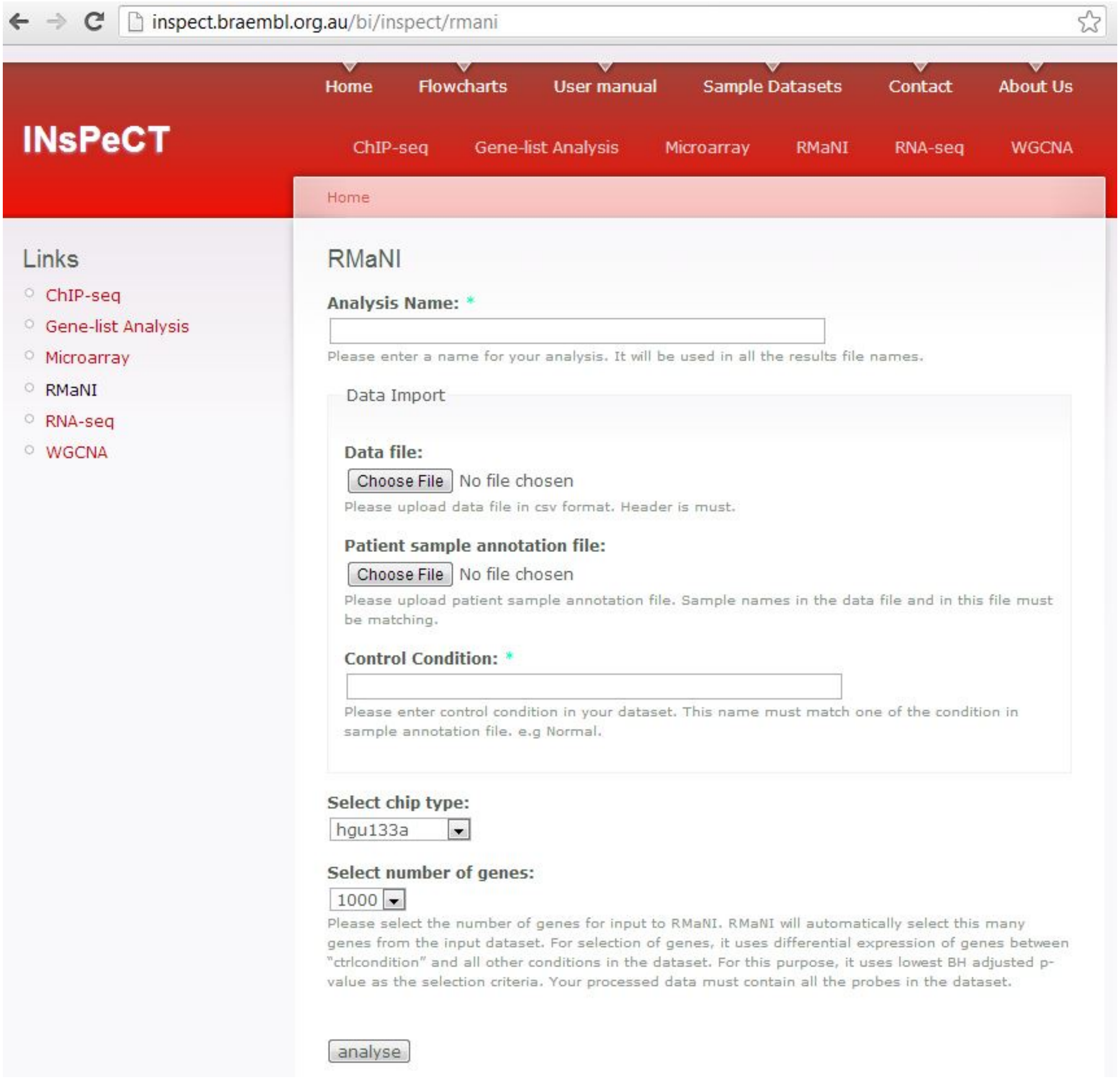

Figure 1 Screenshot of RMaNI homepage.

II. Data Submission Page

Step 1 – Analysis Name

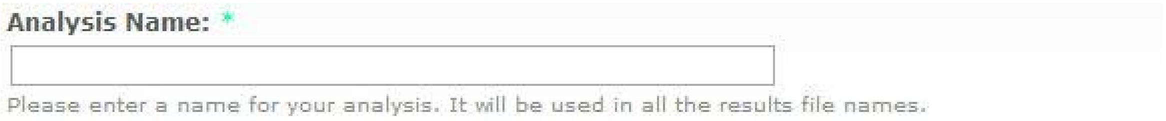

Figure 2 Screenshot of RMaNI's analysis name text box.

This field allows you to enter a short name for your analysis. It is important to enter the name for your analysis because the generated result files will include this name as part of the file names for your convenience. For instance, if you enter "liver" in this field generated files will look like "results_liver.csv". This field is must.

Step 2 – Data Import

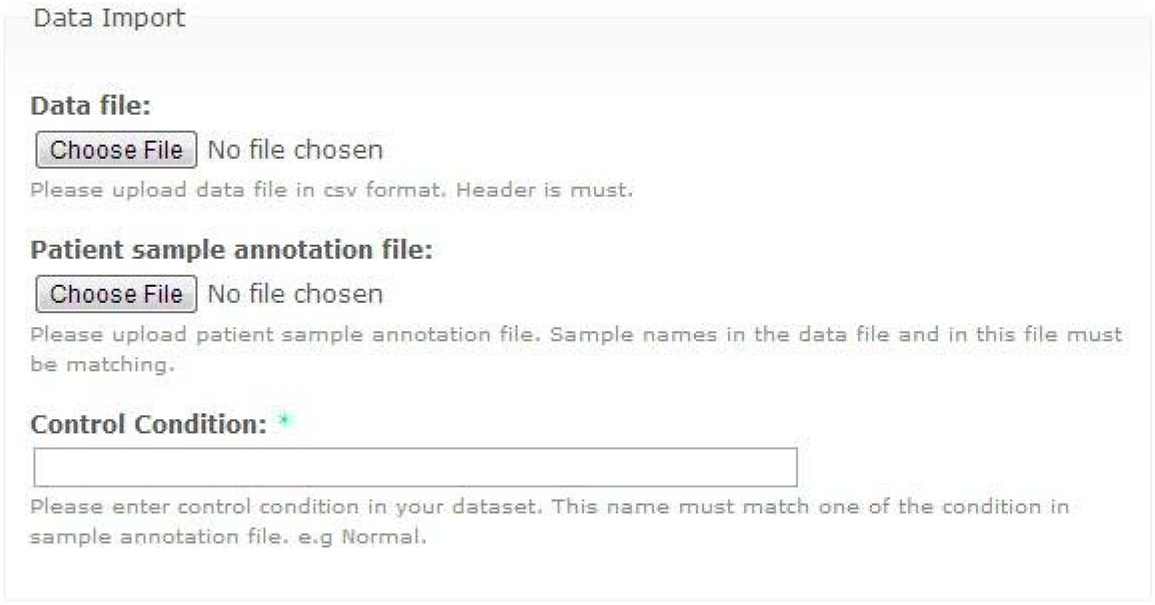

Figure 3 Screenshot of RMaNI's data import section.

Step 2a – Data file upload

Data file upload box in the data import option will allow you to import the pre-processed (background corrected and normalised) microarray gene expression data from your local

computer. File must be in. “CSV (Comma delimited) (*.csv)” format with column header. To convert different file formats to “.csv” format, file format conversion tools are widely available e.g. “ConvertXLS” and “File Conversion Wizard”. Data header’s first column name must be “probe” with probe ids as identifiers and remaining columns must be samples. For file format, please refer the example dataset provided in the sample datasets section. Snapshot of the processed data –

```
probe,GSM359972,GSM359973, GSM359984, GSM360039
117_at,4.9038,6.9663,4.3324,6.8817
1255_g_at,3.6934,4.882,7.1288,6.9821
1438_at,3.2685,3.9953,6.6480,3.4728
```

Step 2b – Patient sample annotation file upload

“Patient sample annotation file” allows you to upload sample annotations. File must be in. “CSV (Comma delimited) (*.csv)” format with column header. To convert different file formats to “.csv” format, file format conversion tools are widely available e.g. “ConvertXLS” and “File Conversion Wizard”. First column name must be “samples” containing sample names exactly matching the samples names in the data file and second column name must be “condition” (like Normal/cancer etc). This is very important for all the downstream analyses because RMaNI will require information about which samples belongs to e.g. “Normal” condition and which samples belongs to different subtypes/conditions e.g. “HCC”, “cirrhosisHCC” or “cirrhosis”. For file format, please refer the example sample annotation file provided in the sample datasets section. Snapshot of the sample annotation file -

```
samples,condition
GSM358209,Normal
GSM358210,Normal
GSM358114,HCC
GSM358115,HCC
GSM358113,cirrhosisHCC
GSM358116,cirrhosisHCC
GSM358119,cirrhosis
GSM358123,cirrhosis
```

Step 2c - Control Condition

For this analysis you must enter the name of the control condition from your sample annotation file eg. “Normal” in the control condition text box. This field is must for comparison of different subtypes/conditions with control condition. If this information is not provided then the analysis will fail. For the sample dataset provided in this tutorial, control condition is “Normal”.

Step 3 - Select chip-type

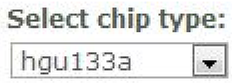

Figure 4 Screenshot of RMaNI’s select chip-type section.

“Select chip type” allow you to select chip-type of your dataset. This is very important as it will affect the annotation of the probes to other gene identifiers and downstream analyses. Please confirm the chip-type of your data and select appropriate option from the list of 13 Affymetrix chip-types. Chip type information can generally be obtained from the meta-information available with the dataset. If the dataset is publicly available, this chip-type information can be available from the repositories e.g. GEO or ArrayExpress. Providing wrong chip-type will fail your analysis or it may output incorrect results.

Step 4 - Select number of genes

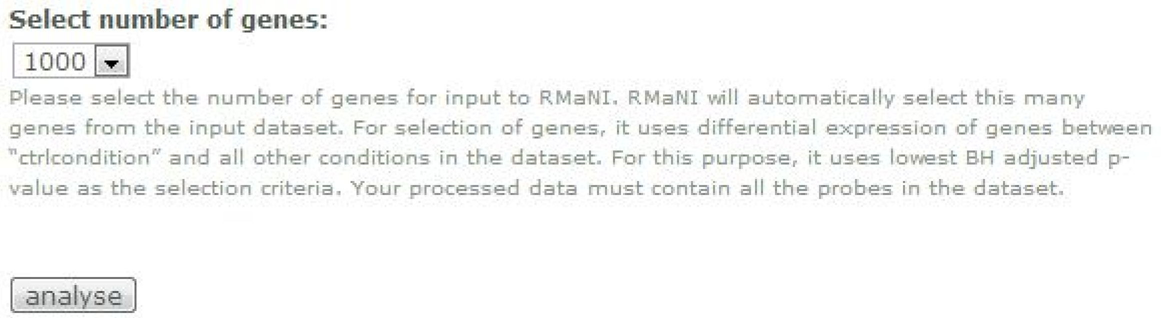

Figure 5 Screenshot of RMaNI's select number of genes and analyse button section.

Here you can select the number of genes for input to RMaNI. RMaNI will automatically select this many genes from the input dataset. For selection of genes, it uses differential expression of genes between "ctrlcondition" and all other conditions in the dataset. For this purpose, it uses lowest BH-adjusted p-value as the selection criteria. Your processed data must contain all the probes in the dataset e.g. ~54000 probes in a dataset arising from "hgu133plus2" chip-type.

Figure 6 shows screenshot of the job submission page after all the required files are uploaded and fields are filled. Once dataset and sample annotation file is uploaded and required fields are filled, you can submit the analysis by clicking analyse button at the bottom of the page.

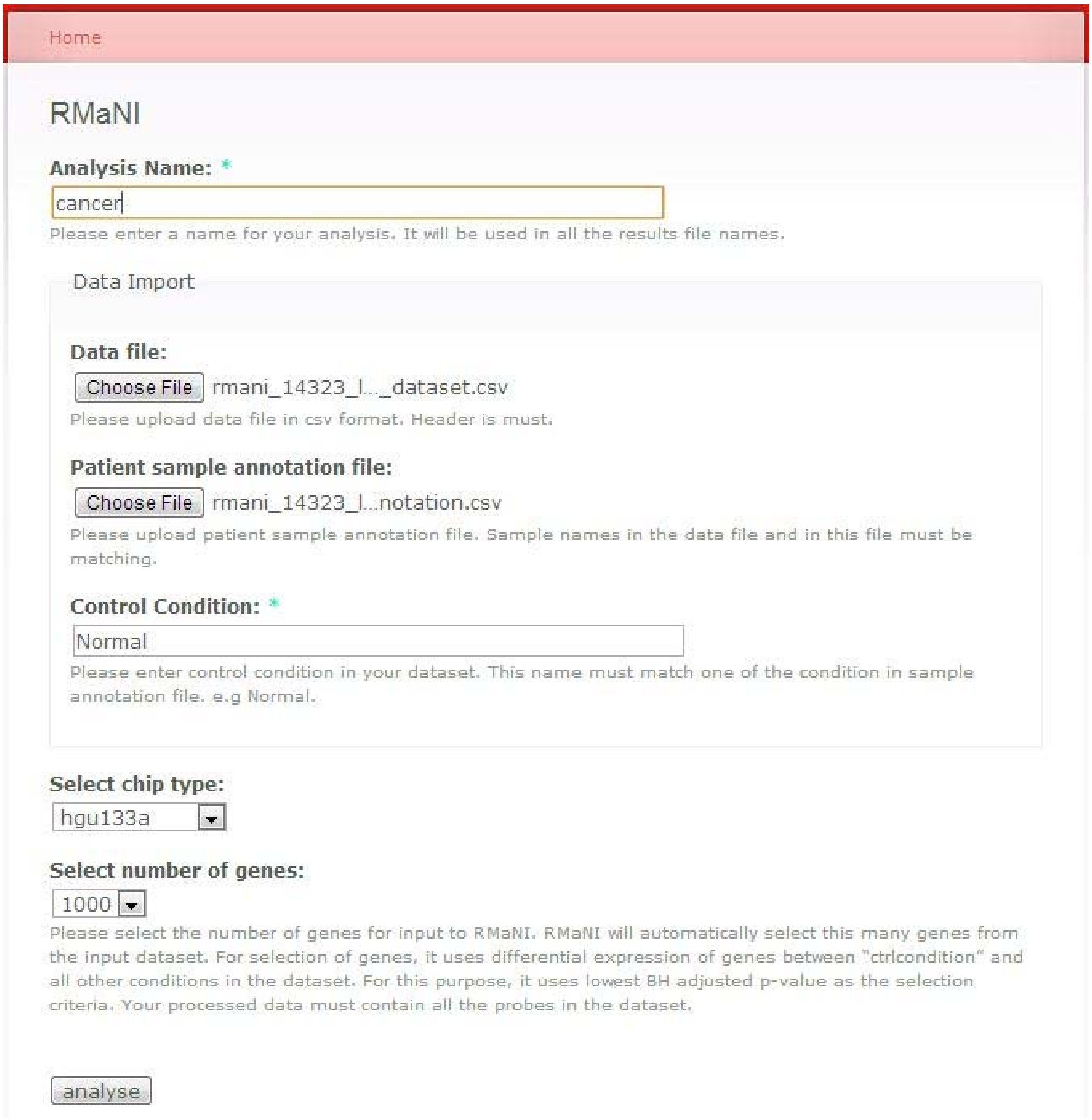

Figure 6 Screenshot of RMaNI's data submission page after all the required files are uploaded and fields are filled.

III. Job submission confirmation, job progress and results

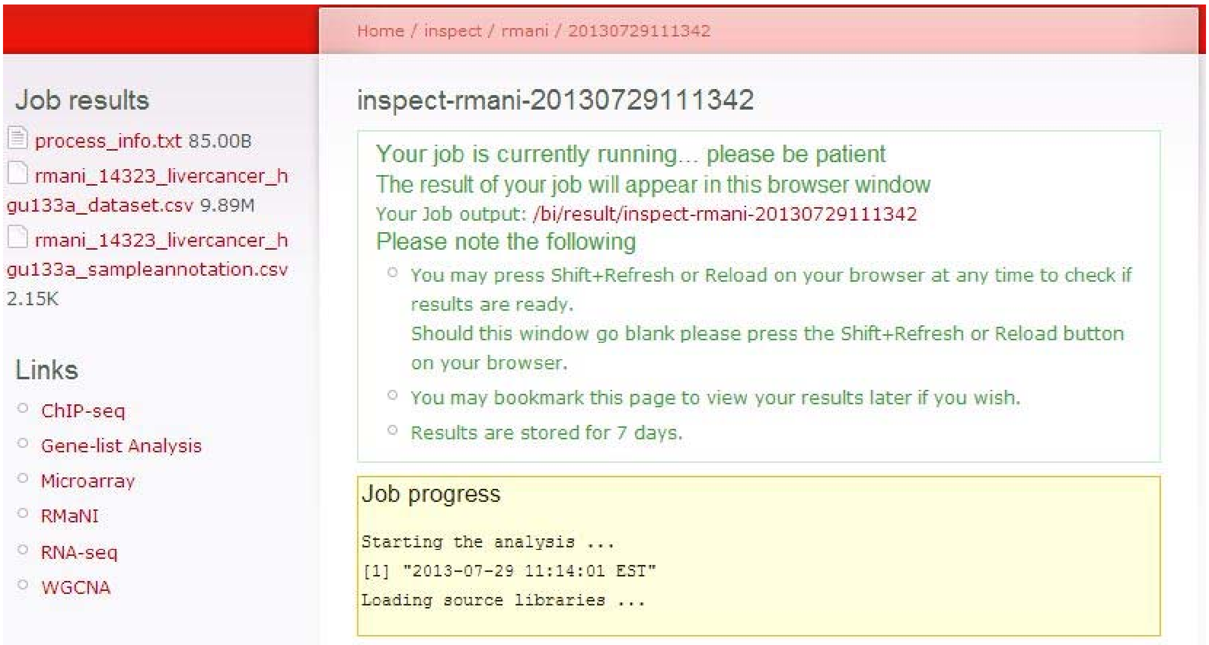

Figure 7 Screenshot of RMaNI's Job submission confirmation, job progress and results section.

Figure 7 shows the screenshot of RMaNI's Job submission confirmation, job progress and results section Once "analyse" button is clicked, job submission confirmation page with unique job id will appear. Detailed job progress will be displayed in "job progress" section of the page. Some steps in the analysis takes longer time than others, in such cases it will display messages such as "This step takes very long time. Please be patient". In the meantime, you may reload your browser at any time to check if results are ready. Depending on the dataset size, analysis can take up to several days to complete. Once the job is finished, results are stored on the server for 7 days. If in case you have closed your browser, you can retrieve your results using the job output link provided. For convenience and simultaneous investigation of your analysis results, "job results" section provides access to result files generated throughout the analysis was being carries out. You can download any of the generated files while the job is running. We also provide access to 'R objects - .rda file' for every step of the analysis. You can download generated R objects and load it in R for further

analyses. Once the job is finished, all the results files can be downloaded as a single ".zip" file. Figure 8 shows screenshot of the page after a job is finished and results are ready.

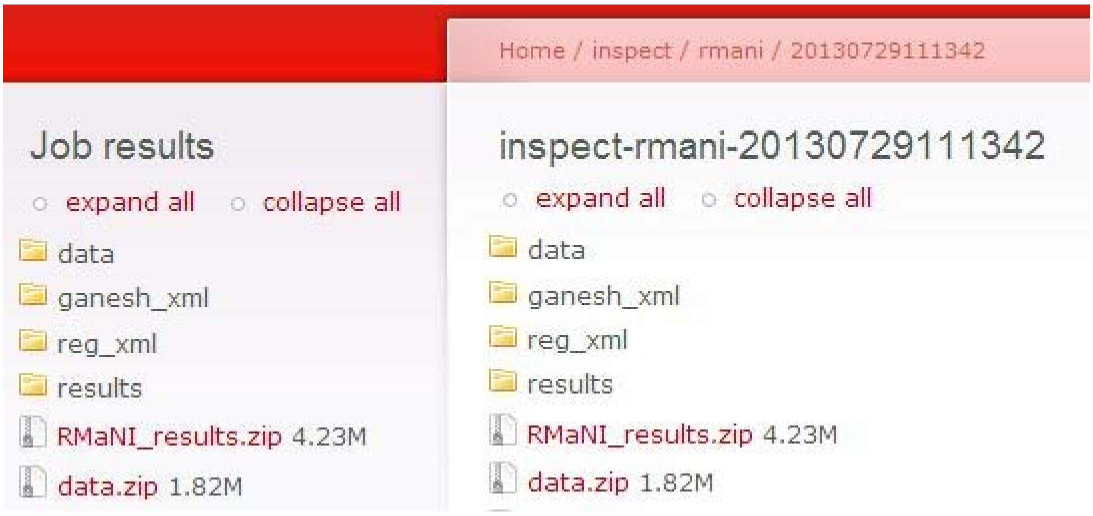

Figure 8 Screenshot of RMaNI's job results page. Results can be downloaded as single ".zip" file or separately from individual folders.
